# Overlapping Genetic Background of Coronary Artery and Carotid/Femoral Atherosclerotic Calcification

**DOI:** 10.3390/medicina57030252

**Published:** 2021-03-09

**Authors:** Anita Hernyes, Marton Piroska, Bence Fejer, Laszlo Szalontai, Helga Szabo, Bianka Forgo, Adam L. Jermendy, Andrea A. Molnar, Pal Maurovich-Horvat, Gyorgy Jermendy, Bela Merkely, David L. Tarnoki, Adam D. Tarnoki

**Affiliations:** 1 Medical Imaging Centre, Semmelweis University, 78/A Üllői Street, 1083 Budapest, Hungary; hernyes.anita@med.semmelweis-univ.hu (A.H.); piroskamarton94@gmail.com (M.P.); bence.fejer@gmail.com (B.F.); laszlo.szalontai.med@gmail.com (L.S.); szabo.helga.se@gmail.com (H.S.); maurovich-horvat.pal@med.semmelweis-univ.hu (P.M.-H.); tarnoki4@gmail.com (D.L.T.); 2 Medical Centre Hungarian Defence Forces, Central Radiological Diagnostic Department, 44 Róbert Károly Boulevard, 1134 Budapest, Hungary; 3 Department of Radiology, Faculty of Medicine and Health, Örebro University, Södra Grev Rosengatan, 70185 Örebro, Sweden; fbia021@gmail.com; 4 Heart and Vascular Center, MTA-SE Cardiovascular Imaging Research Group, Semmelweis University, 18 Határőr Street, 1122 Budapest, Hungary; adam.jermendy@gmail.com (A.L.J.); molnandi@gmail.com (A.A.M.); rektor@semmelweis-univ.hu (B.M.); 5 3rd Department of Internal Medicine, Bajcsy Zsilinszky Hospital and Clinic, 89-91 Maglódi Street, 1106 Budapest, Hungary; gyjermendy@gmail.com; 6 Hungarian Twin Registry, 29 Erdélyi Street, 1212 Budapest, Hungary

**Keywords:** atherosclerosis, multivessel, heritability, genetic correlation, plaque calcification, twin study, coronary artery calcification

## Abstract

Background and objectives: Multivessel atherosclerosis and its genetic background are under-investigated, although atherosclerosis is seldom local and still causes high mortality. Alternative methods to assess coronary calcification (CAC) might incorporate genetic links between different arteries’ atherosclerotic involvement, however, co-occurrences of coronary calcification have not been investigated in twins yet. Materials and Methods: We assessed the heritability of radio morphologically distinct atherosclerotic plaque types in coronary (non-enhanced CT, Agatston score), carotid, and femoral arteries (B-mode ultrasound) in 190 twin subjects (60 monozygotic, 35 dizygotic pairs). Four-segment scores were derived in order to assess the dissemination of the distinct plaque types in the carotid and femoral arteries taking bilaterality into account. We calculated the genetic correlation between phenotypically correlating plaque types in these arteries. Results: CAC and dissemination of calcified plaques in the carotid and femoral arteries (4S_hyper) were moderately heritable (0.67 [95% CI: 0.37–1] and 0.69 [95% CI: 0.38–1], respectively) when adjusted for age and sex. Hypoechoic plaques in the carotid and femoral arteries showed no heritability, while mixed plaques showed intermediate heritability (0.50 [95% CI: 0–0.76]). Age and sex-adjusted phenotypic correlation between CAC and 4segm_hyper was 0.48 [95% CI: 0.30–0.63] and the underlying genetic correlation was 0.86 [95% CI: 0.42–1]. Conclusions: Calcification of atherosclerotic plaques is moderately heritable in all investigated arteries and significant overlapping genetic factors can be attributed to the phenotypical resemblance of coronary and carotid or femoral atherosclerotic calcification. Our findings support the idea of screening extracoronary arteries in asymptomatic individuals. We also propose a hypothesis about primarily carotid-coronary and femoral-coronary atherosclerosis as two distinct genetic predispositions to co-localization.

## 1. Introduction

Coronary artery calcification (CAC) is 50–60% prevalent in asymptomatic middle-aged individuals [1,2] and the prevalence of vascular calcification increases with age, affecting 80% of those aged over 80 years [3]. Even among low-risk middle-aged women, 33% had CAC, which finding improved their cardiovascular (CV) risk prediction [4].

Current guidelines recommend consideration of CAC screening among patients at low-intermediate 10-year CV risk [5], and in cases when statin therapy indication is uncertain [6]. The finding, that individuals with a high clinical score and 0 coronary artery calcification score (CACS) show better survival than individuals with lower risk but positive CACS [7] highlights the importance of this method. The cost-effectiveness of coronary CT as a routine screening tool is still questionable; besides, it involves non-negligible radiation exposure. Thus, experts claim that only a selected group of patients would benefit from CAC screening [8]. Although CAC as a monovascular disease is rare—in the PESA study its prevalence ranged between 11% and 2% showing a gradual decrease in the older age groups [9]—the multi-territoriality of atherosclerosis has not been considered in most studies.

Carotid atherosclerosis had been mainly associated with a higher risk of cerebrovascular events; however, growing evidence suggests a correlation with coronary atherosclerotic involvement [10]. Carotid intima-media thickness, presence of carotid plaque, or total carotid plaque area were found to be also associated with a higher prevalence of adverse coronary events [11,12,13].

On the other hand, the role of femoral ultrasound besides imaging of the carotids in assessing subclinical atherosclerosis and CV risk has also been highlighted recently [14,15]. The CAFES-CAVE study established in 10,000 individuals that prediction of future events was best using a combination of femoral and carotid findings [16]. The AWHS study showed the area under curve (AUC) of receiver operating curve (ROC) of traditional risk factors to predict positive CAC increased significantly from 0.66 to 0.72 when carotid and femoral plaque information was added [17]. Other studies also established a correlation between the carotid-femoral ultrasound findings and coronary artery calcification [18,19,20].

Overall, few studies have investigated the association between different arterial segments’ atherosclerotic burdens so far, and even fewer studies have investigated the genetic background of different arterial segments’ involvement. Although some site-specific genes for femoral artery and aorta were discovered (but not for carotid artery) [21], our previous twin study found that co-occurrence of carotid and femoral atherosclerosis is mainly genetically determined [22]. Plaque co-occurrences of coronary calcification have not been investigated in twins yet.

In the present study, we hypothesized that a carotid-femoral ultrasound-derived trait could well represent the severity of larger-vessel atherosclerosis, which would correlate with the severity of atherosclerosis in the coronaries (as expressed by the Agatston-score) and a common or overlapping genetic risk would underlie this association, supporting the idea that screening other more easily accessed extracoronary arteries might help find asymptomatic individuals at higher CV risk.

## 2. Materials and Methods

### 2.1. Study Participants

The present study is a sub-study of the BUDAPEST—GLOBAL (“Burden of Atherosclerotic Plaques Study in Twins—Genetic Loci and the Burden of Atherosclerotic Lesions”) Study. Detailed study description and enrollment criteria have been published in a previous article [23]. The total study population included 101 Caucasian adult asymptomatic twin pairs recruited from the Hungarian Twin Registry [24]. Study interval was between April 2013 and July 2014. Among all participants, 122 were monozygotic (MZ) and 80 were same-sex dizygotic (DZ) twin subjects. All the participants gave written informed consent. The study has been approved by the National Scientific and Ethics Committee (institutional review board number: ETT TUKEB 58401/2012/EKU [828/PI/12], Amendment-1: 12292/2013/EKU [165/2013] and was consummated according to the principles of the Declaration of Helsinki.

Self-reported questionnaires, physical examinations and non-contrast cardiac CT were performed on day 1, followed by vascular ultrasound on the consecutive day. Due to missing radiological data, we excluded six twin pairs resulting in a final total number of 190 participants.

The data are not publicly available due to lack of consent from participants regarding the publication of their data.

### 2.2. Coronary Artery Calcification—Cardiac Non-Contrast Computed Tomography

ECG triggered, 256-slice multidetector CT with 2.0 mm slice thickness was used during one single inspiratory breath-hold covering 78% of the R-R interval (Brilliance CT, Philips HealthTech, Best, The Netherlands). Depending on BMI, current intensity was set between 20 and 50 mAs, tube voltage was set at 120 kVp. In case of heart rate above 65/min, participants were administered per os *β*-blockers (metoprolol, maximum dose: 100 mg) one hour before the CT scan. The calcification score was determined using commercially available software (Extended Brilliance Workspace, Philips Healthcare, Best, The Netherlands) and expressed using the Agatston score [25].

### 2.3. Vascular Ultrasound

Two expert radiologists with at least 8 years’ experience performed ultrasound examinations using high frequency (5–10 MHz) linear transducers (Philips HD15, Philips Healthcare, Best, The Netherlands). Carotid arteries were scanned bilaterally between the origin of the common carotid artery and the proximal 2–3 cm segments of the internal and external carotid arteries. Common femoral arteries were followed also bilaterally from the level of the inguinal ligament until the bifurcation. Deep femoral and superficial femoral arteries were visualized 1–2 and 3–4 cm long. Plaque definition was the following: endoluminal protrusion of at least 1.5 mm or focal thickening >50% of adjacent intima-media layers. Each detected plaque was described and categorized by their echogenicity type. Taking bilaterality into account, we used 4-segment scores regarding the occurrence in the carotid and femoral arteries regarding and not regarding plaque types.

On the basis of echogenicity, we differentiated hypoechoic, hyperechoic, and mixed plaque-type as described previously [26]. These three categories have been previously validated against histology of carotid endarterectomy specimens: increasing levels of echogenicity correlated with increasing amounts of calcification in endarterectomy plaques; thus, hyperechoic plaque refers to calcified type [27].

### 2.4. Statistics

We performed a descriptive analysis of the questionable variables and relevant patient parameters using SPSS Statistics 17. MZ and DZ groups were compared to each other. We conducted independent samples t-tests in the case of parametric variables, Chi-square tests in the case of binary variables and Mann-Withney U test in the case of nonparametric numeric data. Significance level was set at *p* < 0.05. We demonstrated the co-occurrences of carotid, femoral, and coronary plaques on a Venn-diagram. The relationship between CAC severity and ultrasound findings (plaque location and generalized state) were summarized. Plaque localizations in CAC concordant and discordant MZ and DZ twins were also listed. CAC negative twins were also categorized into two groups: in the first group at least one of the twins had carotid and/or femoral plaque—seen on ultrasound—and in the second group both twins lacked any carotid or femoral atherosclerotic plaque. We performed a non-adjusted Pearson correlation between the four-segment variables and CAC (SPSS Statistics 17).

For twin modeling, we used the OpenMx library for R [28]. The rationale is to compare MZ (sharing ~100% of their genes) and DZ (sharing ~50% of their genes) twins regarding similarity of their traits. Using structural equation modeling, we can estimate the relative importance of the underlying latent factors contributing to the development of a trait: additive genetic factors (A), common environmental factors (C), and unique environmental factors (E) from which the abbreviation ACE is derived [29].

Traits were age and sex-adjusted (as standard practice in quantitative genetic modeling) [30]. Agatston score of CAC was transformed as ordinary variable as follows: 0: no calcification; 1: mild calcification (Agatston score: 1–100); 2: moderate calcification (Agatston score: 101–400); 3: severe calcification (Agatston score > 400). First, we calculated univariate ACE models to assess the heritability of chosen traits using liability-threshold structural equation modeling. Full (ACE) and reduced (AE, CE, E) models were fitted to our data to select the most appropriate (most parsimonious) one. The full model was rejected when one parameter (A, C, or E) could be excluded without significant worsening of the fitness. Each reduced model was compared to the full model by the Chi-square difference test and the best fitting model was chosen according to the Akaike’s Information Criterion (AIC).

Age and sex-adjusted polychoric phenotypic correlations of CACS and 4S_hyper were calculated. Age and sex-adjusted bivariate ACE modeling (correlated factors model) was used for the genetic vs. environmental decomposition of phenotypic similarity using a liability-threshold structural equation model. Model selection was similar to the univariate case, full and reduced models were fitted and compared.

## 3. Results

### 3.1. Descriptive Statistics

Our study cohort included 190 asymptomatic adult twins (120 monozygotic, 70 dizygotic), representing a moderately overweight, middle-aged Caucasian population with a slight female predominance (Table 1). DZ twins were significantly (*p* = 0.01) older and had femoral plaques slightly more frequently (*p* = 0.03) compared to MZ twins. Otherwise, there was no significant difference between MZ and DZ twin subjects regarding classical risk factors and the investigated plaque characteristics.

### 3.2. Plaque Localization

**Figure 1:**
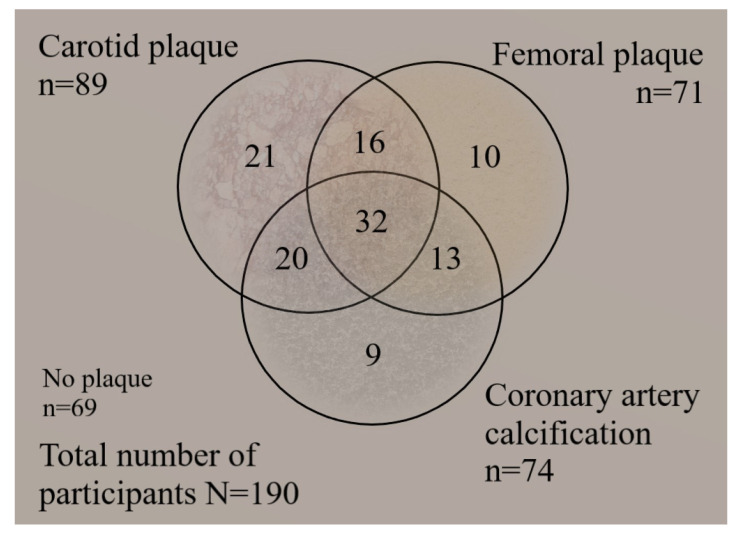
The Venn-diagram showing frequency of overlaps between atherosclerosis localizations in our study participants.

Figure 1 shows that coronary artery calcification alone was the rarest phenomenon. The vast majority of CAC co-occurred with carotid and/or femoral atherosclerosis (87.8%). Femoral-coronary plaque co-occurrence without carotid manifestation was 17.6% of people having CAC. Other 27.0% had carotid-coronary plaque co-occurrence without femoral manifestation, and 43.2% had more generalized atherosclerosis status, affecting carotid, femoral, and coronary arteries. Of the participants, 36% showed no sign of atherosclerosis.

The detailed plaque locations of the MZ and DZ twin pairs, including bilaterality of carotid or femoral atherosclerosis, can be found in Appendix A (Table A2, Table A3, Table A4 and Table A5, Appendix A).

### 3.3. Phenotypic Correlation

Table A1 in Appendix A below summarizes the relationship between the severity of CAC and the dissemination of atherosclerosis seen on ultrasound as expressed by four-segment scores and frequency distribution of different plaque locations (Table A1, Appendix A).

Here we demonstrate the results of the non-adjusted Spearman correlation between CACS (as expressed by the Agatston score) and the four-segment scores based on the ultrasound findings (Table 2).

The four-segment score of hypoechoic plaques showed the weakest correlation (R = 28.9%, *p* < 0.01). In contrast, the combination of mixed and hyperechogenic plaques’ distribution showed the strongest correlation (R = 60.4%, *p* < 0.01), which also seems to be slightly superior to the distribution of any plaque type (R = 55.7%, *p* < 0.01). These results suggest that the dissemination of hypoechoic plaques somewhat weakens the correlation with CACS.

### 3.4. Univariate Analyses

Results of the univariate and bivariate twin statistics are demonstrated in Table 3 and Table 4.

Age and sex-adjusted univariate analyses showed moderate heritability of both CAC (67%) and 4S_hyper (69%). On the contrary, we found that the dissemination of hypoechoic plaques is mainly influenced by unique environmental factors. The 4-segment score of mixed plaque type is 50% heritable and 50% influenced by unique environmental factors.

### 3.5. Bivariate Analysis

The bivariate analysis of CAC and 4S_hyper showed that genetic effects are responsible for the correlation between the calcification of the coronaries and hyperechoic plaque dissemination in the carotid and femoral arteries in 86% with a 95% confidence interval ranging between 42% and 100%. Age and sex-adjusted polychoric phenotypic correlation was 48%, which was slightly higher in the MZ twins than the DZ twins (54% vs. 44%, respectively).

## 4. Discussion

The severity or dissemination status of atherosclerosis in larger arteries as expressed by a four-segments score correlated well with coronary calcification (0.557 *p* < 0.01). Heritability of distinct plaque types showed strongest genetic effects when calcification was present (CAC: 0.67 [95% CI: 0.35–1], 4S_hyper: 0.69 [95% CI: 0.38–1]), while mixed or hypoechoic plaques showed less heritability. We chose to calculate genetic correlation between the highly heritable calcified plaques and found that the age and sex-adjusted contribution of common or overlapping genetic factors was 86% (95% CI: 42–100%).

Previous studies also established an association between carotid-femoral and coronary atherosclerosis and good prediction could be achieved [18,19,20,31]. However, to our knowledge, we are the first group to use four-segment scores (similar to the number of affected territories used earlier) [18]. This ordinary variable correlated well with the ordinary expression of coronary calcification and when we broke down four-segment scores to plaque types, hyperechoic (calcified) plaque scores showed similarly strong correlation (0.551 *p* < 0.01), while hypoechoic (non-calcified) plaques showed weaker (0.289 *p* < 0.01) correlation. Our findings are in line with previous studies about radiological atherosclerosis phenotypes across the arterial tree. Intra-individual differences in plaque composition between carotid and femoral atherosclerotic lesions were observed by MRI with the exception of max % calcification, which was similar in the two arterial beds [32]. A greater extent of similarity between carotid and coronary plaque composition was observed, highlighting that presence of mixed coronary plaque might be suggestive of co-occurring high-risk carotid plaque. The area under curve (AUC) of calcified coronary plaque score for predicting carotid calcification was 0.75 [33]. Arad et al. also found a significant correlation between CAC and carotid calcification score [34].

- Coronary artery calcification’s co-occurrence with atherosclerosis in other arterial sites is less investigated. The PESA study found among 849 asymptomatic individuals that CAC co-occurred with atherosclerosis at other sites (carotid, aorta, and/or iliofemoral regions) in 89% on average [9]. Among our study participants, coronary calcification co-occurred with atherosclerotic plaques in the carotid and/or femoral arteries in 87.8% in close agreement with the previous result. About more generalized arterial calcification one study found that calcification of the superior mesenteric artery was significantly associated with the calcification of five other arterial territories (celiac trunk, coronaries, thoracic aorta, abdominal aorta, and iliac arteries) on CT [35]. Another study found that more than two-thirds of patients over 70 years old showed generalized arterial calcification in all investigated arteries (carotid, coronary, aorta, iliac arteries) and calcified atherosclerotic plaques significantly correlated in different vascular beds [36]. One-by-one carotid-coronary, femoral-coronary, and carotid-femoral atherosclerosis correlations were found to be weaker [37].

To disentangle the seemingly intricate approach of using carotid and/or femoral artery atherosclerosis involvement as four-segment scores and not forget to treat them as different entities we broke down plaque localization and described co-occurrences in each twin participant. We categorized them as CAC concordants and discordants (Table A2, Table A3, Table A4 and Table A5, Appendix A), MZ and DZ twin pairs. As coronary calcification itself is mainly heritable (67% in our study), CAC discordant monozygotic twins were relatively few and the difference between their Agatston score was obviously lower than in the DZ group. The 0 CAC twins in the MZ group also showed milder peripheral atherosclerotic involvement. The biggest difference was between the CAC concordant MZ and the CAC discordant DZ group. CAC concordant MZ twins had similar loci regarding plaque co-occurrences (either predominantly carotid-coronary or femoral-coronary). In the DZ discordant group atherosclerosis could be similarly severe but plaque localizations didn’t overlap much. A study investigating systemic arterial calcification however found no group of people with only carotid and coronary calcification co-occurring under 50-year-old age. CAC rather presented with aortoiliac calcification or more generalized atherosclerosis [36]. This discrepancy might be due to our lack of information about aortoiliac arteries or different study population characteristics.

Other studies investigating heritability also found a moderate contribution of genetic factors in the case of CAC (41.8–43.5%) in a family study [38]. Another family study found the progression of CAC also to be a highly heritable trait [39]. Heritability of carotid plaque presence, area, and composition were found to range between 66 and 78% [40], whereas heritability of femoral atherosclerotic plaques was estimated at 50% with a magnitude of 77% common genetic influence on carotid and femoral plaque co-occurrence [22] which is comparable to our results. The supposed genetic background of coronary calcification includes insertion-deletion polymorphism of the ACE (angiotensin-converting enzyme) gene [41], ApoE (apolipoprotein E) ε3/2 ε3/3 ε4/3 genotypes interfering with the effect of traditional risk factors [42], MMP3 (matrix metalloproteinase-3) genotype influencing the amount of CAC [43], polymorphisms in the gene encoding MGP (matrix ɣ-carboxyglutamic acid protein) [44], and TREML-4 (triggering receptor expressed on myeloid cells) gene encoding fine-tuning inflammatory responses [45]. The Tampere Vascular study investigating systemic and arterial bed-dependent atherosclerosis found site-specific genes of aortic and femoral but not of carotid plaques. Hundreds of genes were found to be up or downregulated, including inflammatory cells [21]. Common genetic loci were found for aortic, carotid, and coronary calcification (3 SNPs); furthermore, serum lipids were found to have an overlap in the genetic predisposition [46]—also supporting our findings. Also, protective genotypes had been proposed, such as GRB2-associated-binding protein 2 (GAB2) and chemokine receptor-2 (CCR-2) polymorphisms which are more frequent in African-Americans and are associated with less calcification—which is also a well-reported difference [47,48]. Further genetic research can help elucidate the relationship between CAC and the calcification of carotid and femoral atherosclerotic plaques.

The relatively small sample size was one of the limitations of our study. Also, we focused primarily on the calcified atherosclerotic plaque phenotype. This infers two hypothetical models: one is that numerosity and dissemination of different plaque types in a cross-sectional twin design could be relevant to different underlying genetic-environmental pathomechanisms regarding each plaque type (distinct heritability values suggest distinct contribution of genes and environment at each stage). This involves that within-individuals, plaques are present at any different stages, each plaque type representing a stage at the continuum of atherosclerosis—and, this cross-sectional sample could catch twin pairs at approximately the same stage of the disease continuum. However, when regarding localization of plaques in twin pairs (see the Appendix A) it rather seems like plaque location is not stochastic, but MZ twins show a higher resemblance, they rather only differ from each other regarding uni- or bilaterality or the overall generalized state of atherosclerosis. This observation suggests a delay of various degrees in atherosclerosis severity within-pair, as for example in the case of the presentation of autoimmune diseases in monozygotic twin pairs [49]. In this cross-sectional study design, however, we cannot conclude any unequivocal inference regarding the longitudinal changes of heritability, such as later and more severe phenotypes are more strongly influenced by genetic factors than earlier ones. Questioning the above hypotheses we also generated new ones when we looked deeper: the genetic resemblance of calcified plaque types observed in our study could be due to an assumed genetic predisposition to plaque location (or “route” of generalization: either primarily carotid-coronary or femoral-coronary) and due to the fact that calcified plaque type is a late, irreversible state of atherosclerosis, giving a higher chance of twin pairs to “gain on” each other, if a time-delay in the presentation of the disease might be observed between them. On the other hand, if different stages of atherosclerosis are indeed related to a different degree of heritability—future genetic research might consider focusing on one specific phenotype regarding atherosclerosis instead of using one surrogate marker. In our asymptomatic twin study population, 36% lacked any sign of atherosclerosis in all investigated arteries and 50–60% lacked atherosclerotic markers regarding the arterial territories one-by-one, which is another limitation. Spearman correlations might show stronger and more significant results—thus, it is recommended to repeat these calculations in a study population where all participants are atherosclerotic. However, in a twin study design the presence of healthy phenotypes are necessary to define the heritability of any given trait.

- Despite the above considerations, our study results strongly support that atherosclerotic calcification is not a passive degeneration that occurs with aging as thought earlier. It is rather an actively regulated process that also might be non-site specific (explaining why intraindividual phenotypic resemblance is high) and highly influenced by genetic predisposition. Generally, there is a growing body of evidence that microcalcification originates from extracellular vesicles released by macrophages and vascular smooth muscle cells within the plaque, which in a collagen-poor environment grow more easily into macrocalcification [50,51,52,53]. Metalloprotease enzymes, developmental, inflammatory, and metabolic factors are thought to regulate the process of atherosclerotic calcification, which in 15–20% can also turn into complex trabecular bone formation due to the plasticity of mesenchymal cells [54,55]. Calcium-phosphate imbalance is also thought to play a role, which contributes to the insufficiency of proposed calcification inhibiting pathways or manifests with the imbalance of positive and negative modulators [56,57]. However, the mechanism of atherosclerotic calcification is still not fully understood—some researchers even suggest that atheroma formation and extensive calcification might be two distinct conditions with some possible overlap [58]. Sage et al. propound that calcification might have an evolutionary explanation: it may be an ultimate immune response mechanism that develops a mechanical barrier [55].
- Screening patients with combined carotid and femoral ultrasound, detection of atherosclerotic calcification could be a cost-effective pre-selecting modality to perform cardiac CT in search for further coronary calcification similarly to the proposal of the PCV METRA group [59]. The common or overlapping genetic background of the calcification process and the hypothesized genetic predilection of plaque locations supports this idea and should generate further prospective research. Although calcified plaques are late, complicated phenotypes, nowadays a wide range of treatment options for heavily calcified plaques are already available such as the double-wire technique and rotational and orbital atherectomy [60]. Also, oral medications are being proposed to treat vascular calcification (primarily in chronic kidney disease)– however, the safety of most of these drugs needs to be addressed, especially in regard of the unwanted parallel inhibition of calcification in bones [61]. Principally, carotid-femoral ultrasound or coronary calcification assessment could help regroup patients regarding their cardiovascular risk better than traditional risk factors do alone. Our study might encourage future investigations about the genetic background of plaque dissemination “route” longitudinally and seek common genetic mechanisms that promote or reduce calcification at these localizations. We believe that a better understanding of the genetic background of atherosclerotic calcification will also lead to better therapeutic options in the future.

## 5. Conclusions

Our study showed major common genetic predisposition to carotid/femoral and coronary atherosclerotic calcification. Phenotypic resemblance and heritability were both high regarding calcified plaque type. Our findings support the idea of combining carotid and femoral ultrasound in the screening of asymptomatic adults, which can help regroup patients regarding their cardiovascular risk or the prediction of coronary atherosclerosis. We also generated the hypothesis that primarily carotid-coronary or femoral-coronary atherosclerosis might be two distinct genotypes, which might generate future longitudinal research.

## Figures and Tables

**Table 1:** Characteristics of the twin participants. ^1^: Four-segment score consists of the number of arterial locations that are affected by atherosclerotic plaque (right/left carotid/femoral artery). MZ: monozygotic twins; DZ: dizygotic twins; 4S_PL refers to any plaque-type detected by ultrasound. ^2^: 4S_hypo refers to the four-segment score of hypoechoic plaques; 4S_mixed refers to the four-segment score of mixed plaques and 4S_hyper refers to the four-segment score of hyperechoic plaques. Hypoechoic plaques contain no calcification, mixed plaques are partly calcified, and hyperechoic plaques are calcified.

	Total	MZ	DZ	P
Zygosity	190	120	70	
Male (*n*, %)	72 (37.89)	48 (40)	24 (34.29)	0.43
Age (mean, SD)	56.84 ± 9.33	55.46 ± 9.75	59.16 ± 8.11	0.01
BMI (kg/m^2^) (mean, SD)	27.57 ± 4.65	27.85 ± 4.46	27.08 ± 4.95	0.27
Hypertension (*n*, %)	79 (41.58)	50 (41.67)	29 (41.43)	0.96
Diabetes (*n*, %)	14 (7.37)	10 (8.33)	4 (5.71)	0.52
Dyslipidaemia (*n*, *%*)	83 (43.68)	49 (40.83)	34 (48.57)	0.26
Smoking (*n*, *%*)	70 (36.84)	44 (36.67)	26 (37.14)	0.89
Coronary plaque occurrence (CACS > 0) (*n*, *%*)	74 (38.95)	44 (36.67)	30 (42.86)	0.39
Carotid plaque occurrence (*n*, *%*)	89 (46.84)	54 (45.00)	35 (50.00)	0.51
Femoral plaque occurrence (*n*, *%*)	71 (37.37)	38 (31.67)	33 (47.14)	0.03
Carotid/femoral and coronary plaque co-occurrence (*n*, *%*)	65 (34.21)	39 (32.50)	26 (37.14)	0.51
Carotid + femoral + coronary plaque co-occurrence (all 3) (*n*, *%*)	32 (16.84)	16 (13.33)	16 (22.86)	0.09
4S_PL > 1 (*n*, *%*) ^1^	119 (62.63)	76 (63.33)	43 (61.43)	0.79
4S_hypo > 1 (*n*, *%*) ^2^	71 (37.37)	46 (38.33)	25 (35.71)	0.72
4S_mixed > 1 (*n*, *%*) ^2^	61 (34.21)	39 (32.5)	22 (31.43)	0.88
4S_hyper > 1 (*n*, *%*) ^2^	75 (37.5)	44 (34.9)	31 (41.9)	0.36
4S_mixed/hyper > 1 (*n*, *%*) ^2^	98 (51.58)	58 (48.33)	40 (57.14)	0.38

**Table 2:** Results of non-adjusted Spearman correlation between coronary artery calcifications score (CACS) and the four-segment plaque scores. 4S_PL: four-segment plaque score as seen on ultrasound (right/left, carotid/femoral involvement) regardless of plaque-type. 4S_hypo: four-segment plaque score of hypoechoic plaques. 4S_mixed: four-segment plaque score of mixed plaques. 4S_hyper: four-segment plaque score of mixed plaques. 4S_mixed/hyper: four-segment score of plaques that are either mixed or hyperechogenic.

N = 190	CACS	*p*
4S_PL	0.557	<0.01
4S_hypo	0.289	<0.01
4S_mixed	0.444	<0.01
4S_hyper	0.551	<0.01
4S_mixed/hyper	0.604	<0.01

**Table 3:** The results of univariate analyses of the investigated traits. Best fitting model is marked with a star (*). AIC: Akaike’s Information Criteria; BIC: Bayesian Information Critera; −2LL: −2 log-likelihood (deviance); df: degree of freedom; diffLL: difference in minus 2*log-likelihoods of the base and comparison models; A: additive genetic effects; C: common environmental effects; E: unique environmental effects.

Trait	Model	Goodness-of-Fit Indices					Parameter Estimates (95% CI)		
Trait	Model	AIC	−2LL	df	DiffLL	*p*-Value	A	C	E
CAC	ACE	337.2	311.9	11	Ref.	Ref.	0.67 (0.16, 1)	0 (0, 0.38)	0.33 (0, 0.67)
	AE *	334.6	311.9	10	0	1	0.67 (0.35, 1)	-	0.33 (0, 0.65)
	CE	340.1	317.5	10	−5.5	.02	-	0.43 (0.15, 0.66)	0.57 (0.35, 0.85)
	E	346.2	326.1	9	−14.1	.00	-	-	1
	Sat.	346.9							
4S_hypo	ACE	408.7	378.2	13	Ref.	Ref.	0 (0, 0.41)	0.18 (0, 0.45)	0.82 (0.55, 1)
	AE	406.9	379.2	12	−0.9		0.13 (0, 0.45)	-	0.87 (0.56, 1)
	CE	406.0	378.2	12	0	1	-	0.18 (0, 0.45)	0.82 (0.55, 1)
	E *	404.9	379.7	11	−1.5	0.47	-	-	1
	Sat.	419.7							
4S_mixed	ACE	342.7	312.2	13	Ref.	Ref.	0.49 (0, 0.76)	0 (0, 0.49)	0.50 (0.24, 1)
	AE *	340.0	312.2	12	0	1	0.50 (0, 0.76)	-	0.50 (0.24,1)
	CE	342.4	314.6	12	−2.4	0.13	-	0.32 (0.02, 0.57)	0.68 (0.43, 0.98)
	E	344.2	319.0	11	−6.8	0.03	-	-	1
	Sat.	355.0							
4S_hyper	ACE	363.4	332.9	13	Ref.	Ref.	0.69 (0.19, 1)	0 (0.38, 1)	0.31 (0, 0.63)
	AE *	360.7	332.9	12	0	1	0.69 (0.38, 1)	-	0.31 (0, 0.63)
	CE	366.6	338.8	12	−5.8	0.02	-	0.41 (0.13, 0.63)	0.59 (0.37, 0.87)
	E	371.9	346.8	11	−13.9	0.00	-	-	1
	Sat.	371.9							

**Table 4:** The results of the bivariate analysis between coronary artery calcification (CAC) and 4-segment hyperechoic plaque score (4S_hyper). Best fitting model is marked with a star (*). AIC: Akaike’s Information Criteria A: additive genetic effect, C: common environmental effect, E: unique environmental effect.

Traits	Adjust	Model	Model Fit (*p*)	Model Fit (AIC)	A	C	E
CAC and 4S_hyper	Age and sex	ACE	-	−81.9	0.99	0	0.01
		AE *	0.98	−87.8	0.86 (0.42, 1)	-	0.14 (0, 0.58)
		CE	0.01	−77.4	-	0.42	0.58
		E	0	−67.1	-	-	1
	Phenotypic correlation						
		All (95% CI)		MZ (95% CI)		DZ (95% CI)	
CAC and 4S_hyper	Age and sex	0.48 (0.30, 0.63)		0.54 (0.31, 0.72)		0.44 (0.14, 0.68)	

## Data Availability

Data available on request due to restrictions eg privacy or ethical. The data presented in this study are available on request from the corresponding author. The data are not publicly available due to lack of consent from participants regarding the publication of their data.

## Appendix A

**Table A1:** Summarizes the relationship between CAC severity and ultrasound findings. Bilateral carotid and femoral plaques were in total 94.11% predictive of positive CAC and 64.7% predictive of moderate or severe CAC. No plaque or unilateral carotid or femoral involvement’s CAC ranged between 87.3 and 50%, however negative ultrasound could rule out moderate or severe CAC in 97.18%. The mean rank of total plaques seen on ultrasound correlates well with severity of atherosclerosis, however a large percentage of people (38.37%) with higher total plaque number than the median had zero CAC score. 4S_PL (four-segment plaque score -any type) and 4S_hypo (four-segment hypoechoic plaque score) showed worse discriminatory power than 4S_mixed (four-segment mixed plaque score) or 4S_hyper (four-segment hyperechoic plaque score). Hyperechoic or mixed plaque score above the median co-occurred with 0 CAC score in 31–33%, thus 67–69% meant positive CAC, and 41–42% meant moderate or severe CAC. The same numbers for hypoechoic or any type of plaque scores above the median are: 35–50% with 0 CAC score, 50–64% with positive CAC score and 30–42% with moderate or severe CAC. However, for ruling out CAC, total plaque number ≤ the median or 4S_PL ≤ the median was better, than the differentiated plaque type scores (78–80% vs. 68–78%).

	CAC Severity			
	0 (*n* = 116)	1–100 (Mild) (*n* = 36)	100–400 (Moderate) (*n* = 22)	>400 (Severe) (*n* = 16)
**Localization of plaques on ultrasound:**				
No plaque/4S_PL = 0/(*n*, *%*)	62 (87.32)	7 (9.86)	2 (2.82)	0 (0.00)
Unilateral Carotid/4S_PL = 1/(*n*, *%*)	20 (68.97)	7 (24.14)	1 (3.45)	1 (3.45)
Unilateral Femoral/4S_PL = 1/(*n*, *%*)	6 (50.00)	5 (41.67)	1 (8.33)	0 (0.00)
Unilateral Carotid and Femoral/4S_PL = 2/(*n*, *%*)	5 (62.50)	2 (25.00)	1 (12.50)	0 (0.00)
Bilateral Carotid/4S_PL = 2/(*n*, *%*)	9 (42.86)	5 (23.81)	4 (19.05)	3 (14.29)
Bilateral Femoral /4S_PL = 2/(*n*, *%*)	1 (20.00)	1 (20.00)	1 (20.00)	2 (40.00)
Bilateral Carotid and unilateral Femoral/4S_PL = 3/(*n*, *%*)	6 (46.15)	2 (15.38)	2 (15.38)	3 (23.08)
Bilateral Femoral and unilateral Carotid/4S_PL = 3/(*n*, *%*)	6 (42.86)	2 (14.29)	4 (28.57)	2 (14.29)
Bilateral Carotid and bilateral Femoral/4S_PL = 4/(*n*, *%*)	1 (0.06)	5 (29.41)	6 (35.29)	5 (29.41)
**Generalized state according to ultrasound findings:**				
Total plaque number (Mean Rank)	73.03	111.00	136.95	163.22
Total plaque number > Median (*n*, *%*)	33 (38.37)	20 (23.26)	17 (19.77)	16 (18.60)
Total plaque number ≤ Median (*n*, *%*)	83 (80.58)	16 (15.53)	4 (3.88)	0 (0.00)
4S_PL > Median (*n*, *%*)	27 (35.06)	17 (22.08)	18 (23.38)	15 (19.48)
4S_PL ≤ Median (*n*, *%*)	89 (78.76)	19 (16.81)	4 (3.54)	1 (0.88)
4S_hypo > Median (*n*, *%*)	35 (49.30)	14 (19.72)	10 (14.08)	12 (16.90)
4S_hypo ≤ Median (*n*, *%*)	81 (68.07)	22 (18.49)	12 (10.08)	4 (3.36)
4S_mixed > Median (*n*, *%*)	19 (31.15)	16 (26.23)	13 (21.31)	13 (21.31)
4S_mixed ≤ Median (*n*, *%*)	97 (75.19)	20 (15.50)	9 (6.98)	3 (2.32)
4S_hyper > Median (*n*, *%*)	24 (32.88)	18 (24.66)	16 (21.92)	15 (20.55)
4S_hyper(≤ Median (*n*, *%*)	92 (78.63)	18 (15.38)	6 (5.13)	1 (0.85)

**Table A2:** Ultrasound findings of MZ (monozygotic) twins concordant and discordant to CAC (coronary artery calcification). CACS: coronary artery calcification score; CAR: carotid artery; FEM: femoral artery; bil.: bilateral; uni: unilateral; o: only. Overlapping localizations are marked with the same colour (orange or blue).

MZ	CAC Concordants:		CAC Discordants:	
	MZ Twin 1+ (More Severe)	MZ Twin 2+	MZ Twin1+	MZ Twin 2−
Mean CACS: 728.65	CAR.bil.o.	CAR.bil.o.	CAR.bil + FEM.bil.	-
	CACS: 1233	CACS: 224.3	CACS: 195.8	-
Mean CACS: 726.25	CAR.bil.o.	CAR.bil. + FEM.uni	CAR.uni.o.	CAR.uni.o.
	CACS: 971	CACS: 481.5	CACS: 19.71	-
Mean CACS: 556.86	CAR.bil. + FEM.uni	FEM.bil. + CAR.uni	-	CAR.uni.o.
	CACS: 822.86	CACS: 290.87	CACS: 10	-
Mean CACS: 533.02	FEM.bil.o.	FEM.bil. + CAR.uni	FEM.bil. + CAR.uni	FEM.uni.o.
	CACS: 675.83	CACS: 390.21	CACS: 7	-
Mean CACS: 499.885	CAR.bil + FEM.bil.	CAR.bil.o.	CAR.bil.o.	CAR.uni + FEM.uni
	CACS: 525.64	CACS: 474.13	CACS: 5.1	-
Mean CACS: 356.04	CAR.uni.o.	CAR.bil.o.	-	-
	CACS: 407.54	CACS: 304.54	CACS: 3.5	-
Mean CACS: 330.28	CAR.bil. + FEM.uni	CAR.bil. + FEM.uni	-	CAR.Bil.o.
	CACS: 466.98	CACS: 193.59	CACS: 2.5	-
Mean CACS: 319.5	CAR.bil. + FEM.uni	CAR.bil. + FEM.bil.	CAR.bil.o.	CAR.uni. + FEM.uni
	CACS: 360	CACS: 279	CACS: 2.23	-
Mean CACS: 254.95	CAR.bil. + FEM.bil.	CAR.uni.	-	FEM.uni.o.
	CACS: 413.58	CACS: 96.32	CACS: 2	-
Mean CACS: 193.35	CAR.uni.o.	FEM.bil. + CAR.uni	-	-
	CACS: 262.2	CACS: 124.5	CACS: 1.11	-
Mean CACS: 138.5	CAR.bil. + FEM.bil.	CAR.bil. + FEM.bil.	FEM.uni.o.	-
	CACS: 195	CACS: 82	CACS: 0.64	-
Mean CACS: 134.225	CAR.bil.o.	CAR.bil.o.		
	CACS: 259.54	CACS: 8.91		
Mean CACS: 124.1	FEM.bil.o.	FEM.bil. + CAR.uni		
	CACS: 209	CACS: 39.2		
Mean CACS: 71.85	CAR.uni.o.	CAR.uni.o.		
	CACS: 91.88	CACS: 51.82		
Mean CACS: 68.465	CAR.bil.o.	CAR.uni.		
	CACS: 80.7	CACS: 56.23		
Mean CACS: 39.5	FEM.uni.o.	FEM.uni.o.		
	CACS: 78	CACS: 1		
Mean CACS: 36	CAR.Bil. + FEM.bil.	FEM.uni.		
	CACS: 68.19	CACS: 3.81		

**Table A3:** Ultrasound findings of MZ (monozygotic) twins without CAC, grouped as: at least one of them has atherosclerotic plaque seen on ultrasound/none of them has. CAC: coronary artery calcification; CACS: coronary artery calcification score; CAR: carotid artery; FEM: femoral artery; bil.: bilateral; uni: unilateral; o: only. Ovelapping localizations are marked with the same colour (orange or blue).

MZ	0 CAC Concordants	0 CAC Concordants		
			0 US Concordants	
	MZ twin 1 (+)	MZ twin 2 (+/−)	MZ twin 1 (−)	MZ twin 2 (−)
	CAR.uni	-	-	-
	CAR.uni. + FEM.uni	-	-	-
	CAR.uni	CAR.uni	-	-
	CAR.uni	-	-	-
	CAR.bil.+ FEM.uni.	FEM.uni.o.	-	-
	CAR.bil.o.	CAR.uni.	-	-
	CAR.uni.	-	-	-
	CAR.bil.o.	CAR.bil.o.	-	-
	CAR.uni.	CAR.uni.	-	-
	FEM.Bil.	FEM.uni.	-	-
	CAR.uni	-	-	-
	CAR.bil.	CAR.uni.	-	-
	CAR.bil.	CAR.uni.	-	-
	CAR.bil.	-	-	-
	CAR.uni.	-		
	CAR.bil. + FEM.uni.	CAR.uni. + FEM.uni.		
	CAR.uni.	-		
	CAR.bil. + FEM.uni.	-		
	CAR.uni	CAR.uni		
	CAR.bilat.fem.uni.	-		
	FEM.uni.	-		

**Table A4:** Ultrasound findings of DZ (dizygotic) twins concordant and discordant to CAC (coronary artery calcification). CACS: coronary artery calcification score; CAR: carotid artery; FEM: femoral artery; bil.: bilateral; uni: unilateral; o: only. Overlapping localizations are marked with the same colour (orange or blue). Contradictory localizations are highlighted with red.

DZ	CAC Concordants:		CAC Discordants:	
	DZ Twin 1+ (More Severe)	DZ Twin 2+	DZ Twin 1+	DZ Twin 2−
Mean CACS: 990.3	CAR.bil. + FEM.bil.	FEM.bil. + CAR.uni	FEM.bil. + CAR.uni	CAR.uni + FEM.uni
	CACS: 1248	CACS: 732.6	CACS: 728.44	-
Mean CACS: 877.5	CAR.bil. + FEM.bil	CAR.uni + FEM.uni	CAR.bil. + FEM.bil.	FEM.bil. + CAR.uni
	CACS: 1467	CACS: 288	CACS: 334.4	-
Mean CACS: 252.85	FEM.bil.o.	CAR.bil. + FEM.bil.	CAR.bil. + FEM.bil.	CAR.uni
	CACS: 413.4	CACS: 92.3	CACS: 202	-
Mean CACS: 221.65	CAR.bil + FEM.bil.	CAR.uni + FEM.uni	CAR.bil.o.	-
	CACS: 438.21	CACS: 5.09	CACS: 196	-
Mean CACS: 170.1	CAR.bil + FEM.bil.	CAR.bil. + FEM.bil.	-	-
	CACS: 256.92	CACS: 83.25	CACS: 189.3	-
Mean CACS: 86.46	FEM.uni	CAR.uni	-	CAR.uni
	CACS: 149.25	CACS: 23.68	CACS: 145.6	-
Mean CACS: 73.2	CAR.bil. + FEM.bil.	CAR.bil. + FEM.uni.	FEM.bil.o.	FEM.bil. + CAR.uni
	CACS: 79.63	CACS: 66.76	CACS: 62.69	-
Mean CACS: 68.74	FEM.bil. + CAR.uni.	CAR.bil. + FEM.uni	CAR.uni.	CAR.bilat + FEM.uni
	CACS: 114.91	CACS: 22.57	CACS: 61	-
			CAR.uni + FEM.uni	-
			CACS: 14.34	-
			-	-
			CACS: 14	-
			FEM.uni	-
			CACS: 11	-
			CAR.bil.o.	FEM.bil. + CAR.uni
			CACS: 6.36	-
			-	CAR.bil. + FEM.uni
			CACS: 3.02	-

**Table A5:** Ultrasound findings of DZ (dizygotic) twins without CAC, grouped as: at least one of them has atherosclerotic plaque seen on ultrasound/none of them has. CAC: coronary artery calcification; CACS: coronary artery calcification score; CAR: carotid artery; FEM: femoral artery; bil.: bilateral; uni: unilateral; o: only. Overlapping localizations are marked with the same color (orange or blue).

DZ	0 CAC Concordants		0 CAC Concordants	
			0 US Concordants	
	DZ Twin 1	DZ Twin 2	DZ Twin 1	DZ Twin 2
	CAR.bil.o.	-	-	-
	CAR.uni.	-	-	-
	FEM.bil. + CAR.uni	CAR.bil. + FEM.bil.	-	-
	FEM.uni	-	-	-
	FEM.bil. + CAR.uni	-	-	-
	FEM.bil.o.	CAR.uni. + FEM. Uni	-	-
